# Lack of Seasonal Variations in Vitamin D Concentrations among Hospitalized Elderly Patients

**DOI:** 10.3390/ijerph18041676

**Published:** 2021-02-09

**Authors:** Justyna Nowak, Bartosz Hudzik, Paweł Jagielski, Karolina Kulik-Kupka, Aleksander Danikiewicz, Barbara Zubelewicz-Szkodzińska

**Affiliations:** 1 Department of Cardiovascular Disease Prevention, Department of Metabolic Disease Prevention, Faculty of Health Sciences in Bytom, Medical University of Silesia, 41-900 Bytom, Poland; bhudzik@sum.edu.pl; 2 Third Department of Cardiology, Silesian Center for Heart Disease, Faculty of Medical Sciences in Zabrze, Medical University of Silesia, 41-800 Zabrze, Poland; 3 Department of Nutrition and Drug Research, Faculty of Health Science, Jagiellonian University Medical College, 31-007 Cracow, Poland; paweljan.jagielski@uj.edu.pl; 4 Department of Nutrition-Related Disease Prevention, Department of Metabolic Disease Prevention, Faculty of Health Sciences in Bytom, Medical University of Silesia, 41-900 Bytom, Poland; karolina_kulik@interia.pl (K.K.-K.); adanikiewicz@sum.edu.pl (A.D.); bzubelewicz-szkodzinska@sum.edu.pl (B.Z.-S.)

**Keywords:** vitamin D, season, elderly people, vitamin D deficiency

## Abstract

**Background**. Generally, most vitamin D in the human body (90–95%) is produced in the skin during exposure to sunlight. The effectiveness of this process depends on several biological and physical factors, e.g., age or latitude. Skin synthesis of vitamin D among elderly people is reduced. The aim of the study was to assess serum 25-hydroxyvitamin D [25(OH)D] seasonal variations in elderly patients hospitalized at the geriatric department. **Methods**. The study was carried out on 242 patients aged 60 years or older hospitalized at the geriatric department. The study group was categorized by four seasons as well as month. **Results**. The median (interquartile range) 25(OH)D concentration among all patients (*n* = 242) was 33.95 (26.96–45.18) nmol/L. There was no statistical significance in the median serum 25(OH)D concentration with regard to each of the four seasons: in the spring 32.95 (25.96–43.68) nmol/L, in the summer 38.69 (27.46–50.67) nmol/L, in the autumn 33.45 (27.08–44.18) nmol/L, in the winter 34.57 (23.46–43.93) nmol/L, (*p* = 0.48). **Conclusions**. Vitamin D deficiency was observed in all geriatric patients, irrespective of the season. The results of the study indicate no significant differences in median vitamin D concentration among the hospitalized patients across all four seasons. Even in the summer months, in our climate, it is fairly difficult for an elderly person to produce an adequate amount of vitamin D through the skin. Therefore, proper vitamin D supplementation is recommended and should be implemented in the elderly irrespective of the season.

## 1. Introduction

Vitamin D is a fat-soluble vitamin but is more like secosteroid hormone [1]. The well-known function of this vitamin is bone and calcium/phosphorous homeostasis as well as musculoskeletal system modulation. The extraskeletal effects of vitamin D in the last few years are still a matter of debate [1]. The active form of vitamin D–1,25-dihydroxyvitamin D_3_ regulates multiple cellular processes (including cell proliferation, differentiation, apoptosis, angiogenesis and immune modulation) as the vitamin D receptors are present in the kidneys, heart and immune cells [2], cancer cells of the prostate, breast and colon [1,3]. Vitamin D deficiency is correlated with the common prevalence of metabolic and cardiovascular diseases (hypertension, type 1 diabetes mellitus, stroke or myocardial infarction), several autoimmune diseases (including multiple sclerosis, inflammatory bowel disease, rheumatoid arthritis, systemic lupus erythematosus) [2,3].

Vitamin D is an umbrella term for two forms. The first form—the vitamin D_2_ (ergocalciferol), is synthesized by ultraviolet B light (UVB) from the ergosterol in yeast and fungi. Vitamin D_2_ in the diet comes from plants and fungi. The second form—vitamin D_3_ (cholecalciferol) is synthesized from 7-dehydrocholesterol in the skin via UVB sunlight exposure and in the diet comes from animal products (e.g., fatty fish—eel, wild salmon, herring, mackerel, sardines, some fish oils-cod liver oil, eggs, dairy products) [1,4]. Most of the vitamin D in the human body comes from skin synthesis. Next, 7-dehydrocholesterol in the skin is converted to pre-vitamin D_3_ (a thermodynamically unstable pre-vitamin D_3_ form) and later to more stable form-vitamin D_3_ (cholecalciferol) [1,3,4]. Vitamin D (either formed in the skin and absorbed from the diet) is transported in the blood by vitamin D binding protein to the liver where it is hydroxylated by vitamin D 25-hydroxylase (CYP2R1) and form 25-hydroxy vitamin D–25(OH)D_3_–calcidiol. This is the major circulating form of vitamin D and the accepted biomarker for vitamin D status [1,3,4]. Finally, 25(OH)D_3_ is transported to the kidneys and hydroxylated by 1-α-hydroxylase (CYP27B1) to form 1,25-dihydroxyvitamin D–1,25(OH)_2_D–calcitriol, which is the biologically active form of vitamin D [1,4]. Many extrarenal tissues, mainly osteoclasts, colon, brain and blood cells such as macrophages, also express this enzyme, and that is why vitamin D can act in a paracrine and autocrine manner in these tissues [5,6].

The effectiveness of vitamin D skin synthesis depends on the biological and physical factors, e.g., skin pigmentation, age, and latitude [7,8]. Physical factors including sunscreens, clothing, glass shielding may markedly reduce or completely eliminate the production of vitamin D in the skin (the use of sunscreen with a sun protection factor of 15 or higher prevents about 99% of dermal vitamin D production) [8]. The skin synthesis of vitamin D during winter months at latitudes above 37°N and below 37°S solar angle is insufficient [8]. Thus, in Poland, skin synthesis does not occur from October to March. Moreover, in Central Europeans, adequate conditions to skin synthesis of vitamin D are described as exposing 18% of the body to the sunlight without sunscreens for about 15 min a day between 10 AM and 3 PM [7].

The biological factors that reduce vitamin D production and bioavailability are skin pigmentation (increased skin pigmentation can reduce dermal produce vitamin D as much as 99.9%), fat malabsorption, body fat content, medication use, and age [7,8,9]. A decreased cutaneous synthesis of vitamin D is reported in elderly people. Aging is associated with a significant decrease of 7-dehydrocholesterol skin synthesis caused by structural changes in the dermis–shrinkage and decreased elasticity of the papillary dermis and a decrease in superficial loops on the papillary body beneath the dermis. Thus, a 70-year-old person produces 75% less vitamin D via skin synthesis compared with a 20-year-old person [8,9]. Lower vitamin D concentrations in elderly people in comparison to younger people are reported throughout the whole year irrespective of the season [8].

Elderly people are at risk of developing vitamin D deficiency caused by various factors such as decreased dietary intake and impaired intestinal absorption, reduced sunlight exposure, impaired skin synthesis, and hydroxylation in the liver and kidneys [4,8,10].

The evidence examining seasonal variations in vitamin D concentration among the elderly is scarce [11]. There are also, to our knowledge, no studies of this type searching for seasonal changes in the same cohort in regions at latitude 50°17’51” N, where sunlight is not adequate throughout the year. Therefore, we have set out to investigate seasonal variations of serum 25(OH)D in elderly patients hospitalized at the geriatrics department.

## 2. Methods

### 2.1. Study Population

The study was carried out with 422 patients over 60 years hospitalized at the geriatric department of a county hospital in Piekary Slaskie, Silesia, located in the south of Poland (50°N). In this region, skin synthesis does not occur from October to March [7]. The data on clinical presentation, baseline characteristics and laboratory examinations were obtained from the electronic medical records. Exclusion criteria included marked physical and/or mental impairment, liver disorders, decompensated thyroid disease, cancer, anticonvulsants or glucocorticosteroid use, vitamin D supplements use within the 3 months prior to admission. Two hundred forty-two elderly individuals met inclusion criteria. The study was conducted in accordance with the Declaration of Helsinki was approved by the Bioethics Committee of the Medical University of Silesia, Katowice, Poland. All the participants provided written informed consent before participating in the study.

### 2.2. Methods

The study was performed in 2013. Venous blood samples were collected after overnight fasting at admission. Samples were centrifuged to separate serum. The laboratory workup was done on each sample on the day of collection. The blood samples were not stored. The study group was categorized by four seasons: spring (*n* = 61, blood samples collected between 21 March to 20 June), summer (*n* = 77, blood samples collected between 21 June to 22 September), autumn (*n* = 64, blood samples collected between 23 September to 21 December), winter (*n* = 40, blood samples collected between 22 December to 20 March) and moths.

### 2.3. Laboratory Measurements

Serum 25(OH)D concentration (nmol/L) was measured using an enzyme-linked immunosorbent assay (ELISA) (Architect 25-OH vitamin D test, detection range: 10–400 nmol/L,% CV: ≤10). Ingested and cutaneously produced vitamin D is rapidly converted to 25(OH)D, but in serum, only a fraction of 25(OH)D is converted to its active metabolite 1,25(OH)2D. Thus, measurement of the total 25(OH)D concentration is the best test to assess body stores of vitamin D. The total 25(OH)D concentration allowed for the diagnosis and monitoring of vitamin D deficiency, whereas quantification of 25(OH)D2 and 25(OH)D3 fractions can facilitate treatment monitoring [12]. In the laboratory, each step of the diagnostic process was constantly monitored and controlled. Measurement was conducted according to a quality management system compatible with the standard EN-PN 9001:2008.

According to the diagnostic threshold defining serum 25(OH)D concentration approved for Central Europe, serum 25(OH)D concentrations ≤50.0 nmol/L were considered as vitamin D deficient, while concentrations between >50.0 nmol/L and 75.0 nmol/L were classified as suboptimal vitamin D status. Concentrations between >75.0 nmol/L–125.0 nmol/L were defined as adequate vitamin D status [7].

### 2.4. Statistical Analysis

A Shapiro–Wilk test was used to evaluate the normality of variables. Normally distributed data were compared using the Student’s t-test or analysis of variance (ANOVA), while nonparametric data were compared using the Mann–Whitney *U* test and the Kruskal–Wallis test, respectively. Analyzed variables are expressed as mean ± SD (for normally distributed data) as well as median, quartile lower and quartile upper (for nonparametric data). A probability level of *p* ≤ 0.05 was considered to be significant. The data were analyzed using the statistical software STATISTICA 13 PL (Tulsa, Oklahoma, OK, USA).

## 3. Results

### 3.1. Characteristics of the Study Group

A total of 242 patients (70 males, 172 females) with a median age of 78.0 (72.0–83.0) years were included in the study. The median age of women was 79.0 (73.0–83.0) years; the median age of men was 77.50 (72.0–83.0) years (*p* = 0.567). Most of the participants in our study were lived in the town (84.7%), while 15.3% were lived in the village. The main characteristics of the study group are shown in Table 1.

### 3.2. Seasonal Variation of Serum 25(OH)D Concentrations

Seasonal variation of serum 25(OH)D concentrations are shown in Table 2. The median serum 25(OH)D concentration among total patients (*n* = 242) was 33.95 (26.96–45.18) nmol/L. There were no statistically significant differences in the median serum of 25(OH)D concentration during seasons (*p* = 0.480).

Most of the participants (*n* = 193; 79.8%) had vitamin D deficiency. Suboptimal vitamin D concentration was observed in 46 (19.0%) patients. Only three patients had adequate vitamin D concentration (these patients were connected to the group patients with suboptimal vitamin D). The proportion of patients with 25(OH)D deficiency was similar between seasons-respectively 83.6% in spring, 72.7% in summer, 81.2% in autumn and 85.0% in winter. The proportion of suboptimal vitamin D concentration was the same, similar across each season, *p* = 0.297 (Table 3).

Table 4 shows the median serum 25(OH)D concentrations for each month. There were no statistically significant differences in the median serum 25(OH)D concentration among respective months (*p* = 0.193).

## 4. Discussion

In physiological conditions, most vitamin D (90–95%) is produced in the skin tissue during sun exposure. Vitamin D concentration is related to many factors such as various unchangeable factors, such as season, time of day, latitude, air pollution, weather conditions, etc., as well as others like time spent outdoor, using sun protection cream, diet, body mass, etc. Vitamin D concentration also depends on age, race, pigmentation, etc. [8]. Season-dependent vitamin D deficiency has been described, especially during the winter [14]. Poland, as a country located in Central Europe on latitude between 49 and 54°N, has vitamin D skin synthesis present mainly between late April and early September. From October to March, skin synthesis of vitamin D does not occur [7].

Diet is the second source of vitamin D. In Poland; it is a significantly less effective source (compared to skin synthesis). Dietary sources of vitamin D include mainly comprise fatty fish (e.g., eel, wild salmon, herring, mackerel, sardines), some fish oils (e.g., cod liver oil) and to a lesser amount-egg yolk, cheese, milk and some mushrooms. This kind of foods (especially oily fish) are consumed in low amount in particular among elderly people. In some countries, a mandatory fortification of selected food products (milk or dairy products, orange juice, margarine, cereals) is provided. This depends on the health policy and governmental strategies of countries. In the United States, some foods are fortified in vitamin D (e.g., milk, some cereals, orange juice, yogurts, margarine). In European countries, mostly margarine and some cereals are fortified. In Poland, food fortification has not been customary. Some suggest shown that is the reason for the pandemic of vitamin D deficiency in Poland. Evaluation of the composition of diet in different populations showed that when an additional source of vitamin D is reduced (skin synthesis), even a varied and balanced diet cannot serve to match the complete vitamin D requirement [8,15]. In our study, we did not assess the dietary intake of vitamin D. We only excluded patients with supplementation of vitamin D three months before being admitted to the hospital.

We have demonstrated that vitamin D deficiency is common among elderly patients (79.80% of patients had vitamin D concentration below or equal to 50.00 nmol/L). There were only three patients who had an adequate vitamin D concentration. Admittedly, we have shown that the proportion of patients with 25(OH)D deficiency was similar in the spring as well as in the summer, in the autumn and in the winter (respectively 83.60%, 72.70% 81.20%, 85.00%), *p* = 0.297. We observed the minimum median serum 25(OH) D concentration in March and the maximum in January. We did not find statistically significant differences in the medians of serum 25(OH)D concentration during seasons (*p* = 0.480) as well as between the respective months (*p* = 0.193).

Risk factors contributing to vitamin D deficiency among the elderly population include, e.g., increasing adiposity [4]. A systematic review and meta-analysis research indicates that the prevalence of vitamin D is more elevated in obese individuals. Vitamin D deficiency is associated with obesity, irrespective of age and latitude. One of the reasons for the relationship between obesity and vitamin D deficiency may be excess body fat that retains the vitamin D metabolites. The cholecalciferol produced by the skin or intake with the diet is partially sequestered by the body fat before being transported to the liver for hydroxylation [16]. In our study, over 70% of patients had excess body weight. There were no statistically significant differences (*p* = 0.348) in the median serum 25(OH)D concentration among group patients with normal weight (31.70 nmol/L), overweight (35.44 nmol/L) and obesity (34.44 nmol/L).

In the current study, we have set out to examine seasonal variations in 25(OH)D concentrations among elderly hospitalized patients, which is a somewhat specific population given the time amount spent indoors (i.e., hospitalization), although we have attempted to mitigate that effect by taking blood samples at admission. Studies show that people mostly staying in an indoor environment (e.g., shift workers and indoor workers) are consistently reported to be at a greater risk for vitamin D deficiency [17,18]. Several studies have described that exposure to sunlight itself in various populations is indeed enough for the serum vitamin D concentrations to increase during the summer. For example, Bozkurt et al. within a healthy population aged between 20 and 87 from Ankara region, Turkey, has shown a higher serum 25(OH)D concentration in the summer-54.62 ± 38.34 nmol/L (*n* = 173) in comparison to the winter-38.34 ± 23.72 nmol/L (*n* = 262), *p* < 0.001. However, 25(OH)D insufficiency was still found in 94% of patients in the winter and 85% in the summer [19]. This high prevalence of vitamin D insufficiency occurs in this region despite the location of the study group (Turkey as a Mediterranean country; 39–56°N). This seasonal difference in serum 25(OH)D concentration in this study population probably results from younger age and health status.

Similar results were observed among the elderly people of Mexican origin (Mexico, 25°N), where the serum 25(OH)D concentration was lower in the winter compared to the summer (*p* < 0.05) and autumn (*p* < 0.001). The lowest serum 25(OH)D concentration-46.92 ± 18.72 nmol/L were observed among the study population in the winter and has risen in the spring to 49.92 ± 18.22 nmol/L when reached its maximum of 52.41 ± 18.22 nmol/L (in summer) and 54.66 ± 19.72 nmol/L (in autumn). Moreover, in this study, the prevalence of vitamin D deficiency and insufficiency were around 90% [11].

Another study by Lewandowski et al. with the same (as our study) geographical location but differs with respect to age and health status considers the mean serum 25(OH)D concentration in healthy Polish (aged 22–63). Summer serum 25(OH)D concentration were higher in comparison to winter serum 25(OH)D concentration (51.87 ± 18.05 vs. 38.99 ± 18.09 nmol/L; *p* < 0.001) [20].

Another study conducted on 153 elderly individuals living in nursing homes in Brazil by Sousa et al. has shown some interesting results [21]. The mean serum 25(OH)D concentration was higher (59.65 ± 41.43 nmol/L) than in our group, but still, most of this study group (71.2%) had vitamin D concentration below the normal range (25(OH)D ≤ 72.38 nmol/L). The study population consisted of elderly patients institutionalized in the region with high UV light exposure.

Cited studies [11,19,21] have shown that even in the countries with high and very high annual average UV indexes like Turkey, Mexico, Brazil, the prevalence of hypovitaminosis D among elderly people is common even during the summer. It could be caused by the fact that elderly people, in particular, try to avoid high temperatures and any sunlight exposure [22]. Another explanation is the fact that elderly people use sunscreen, cover themselves or have health conditions (so they spend more time indoor)), which in turn predispose them to a decreased skin synthesis of vitamin D [22].

Similar results were reported based on the analysis of Geriatric Rehabilitation Facility patients aged <60 years in Germany. In this study, 96% of patients were vitamin D values below 75.0 nmol/L, similar to our study. Among both groups of patients, the concentration(s) of vitamin D was unaffected by the time of the year. Over summer months, mean serum 25(OH)D concentration was 26.70 ± 22.34 nmol/L, while over winter months were 23.19 ± 20.12 nmol/L. Despite high UV exposure over the summer, which should affect the vitamin D skin synthesis (in Poland as well as in Germany), 96% of German patients had vitamin D deficiency [23].

Different results were demonstrated in another study carried out on a regular community-dwelling group of people aged ≥65 also in Germany. It was reported that the serum 25(OH)D concentration was strongly associated with the seasons. The serum 25(OH)D concentration reached a minimum value of 38.44 ± 16.37 nmol/L in March as the same as in our study-30.45 (21.22–34.44) nmol/L. Moreover, serum 25(OH)D concentration reached a maximum value in August—63.89 ± 16.45 nmol/L, whereas, in our study, it was in January—43.56 (23.74–50.67) nmol/L. Unfortunately, in this study, the authors did not analyze the difference in vitamin D values across all four seasons [24].

Some interesting studies tried to investigate if time spent outdoors may change the serum concentration of vitamin D among the elderly. One of them was carried out on a group of patients aged ≤65 in a psychiatric hospital in Norway. Individuals who spent less than 30 min outdoors every day (over the three months period before the study) had the mean serum 25(OH)D concentration at the concentration of 39.49 ± 17.19 nmol/L and those who spent more than 60 min outdoors every day over the same period of time had the mean serum at the concentration of 33.99 ± 14.00 nmol/L. There was no significant difference. Similar to our study, the mean serum concentration of vitamin D was higher in summer, but the differences in concentration of vitamin D between seasons were not statistically significant [25]. Skin production of vitamin D is not efficient for over six months of the year due to the high latitude of the location of the hospital (Tromso in Norway) where the study was conducted.

The prevalence of vitamin D deficiency among patients (aged 19 or older) was described by Ostrowska et al. [26]. Patients hospitalized during the summer had a higher serum concentration of vitamin D in comparison to patients hospitalized during the winter (45.42 ± 28.00 vs. 36.39 ± 27.48 nmol/L, respectively; *p* = 0.001), but still, 93% of the patients hospitalized during the winter and as much as 86% of the patients hospitalized during the summer had serum 25(OH)D concentration below 75.0 nmol/L. The highest mean concentration of vitamin D was observed in June and the lowest in December (*p* = 0.034) [26]. The key factor of the differences between our study and the one mentioned above is younger age.

The seasonal variation of vitamin D among elderly people could not always be observed. Despite the higher concentration of vitamin D during the summer and lower during the winter or the early spring, vitamin D deficiency is still present [11,19,21,23]. It could be explained by an insufficient skin production of vitamin D among elderly individuals during the summer, even if the sunlight exposure exists. First, elderly people usually spend less time outdoors, but even if they go outside, they tend to be more active in the morning or late evening, so the effect of their skin synthesis of vitamin D over this time is reduced due to the insufficient UV light exposure [4,8,10]. Second, it can be reduced even more by using sunscreen or cloths, especially in our temperate climate, and above all, the most important cause of the insufficient skin synthesis of vitamin D is the reduction of 7-dehydrocholesterol concentration in the skin in comparison to each concentration among the group of younger people [8,9]. As it had been described, a 70-year-old person produces 75% less vitamin D via skin synthesis in comparison to a 20 year-one [8,9]. Moreover, it is essential for specialists to remember this fact whenever they work with elderly individuals.

The guidelines for vitamin D supplementation in Poland issued in 2018 [15] are consistent with the guidelines of Central Europe [7], and they take into account seasonal patterns. It is recommended that elderly people supplement vitamin D throughout the whole year. The reason for this supplementation is that the vitamin D is not produced in a sufficient amount even over the summer months [15].

## 5. Study Limitations

This study should be interpreted in view of its limitations. First and foremost, vitamin D status was assessed only once for each of the studied patients as such seasonal variations refer to the studied population, not a specific patient. Second, we lack information on sunlight exposure or sunscreen product use in the period preceding hospitalization–factors, which could have limited the efficacy of UVB exposure. Finally, there is a lack of a control group consisting of a healthy elderly population.

## 6. Conclusions

In conclusion, vitamin D deficiency was observed in all geriatric patients, irrespective of the season. The results of the study indicate no significant differences in median vitamin D concentration among the hospitalized patients across all four seasons. Even in the summer months, in our climate, it is fairly difficult for an elderly person to produce an adequate amount of vitamin D by the skin. Therefore, proper vitamin D supplementation should be recommended and implemented in the elderly irrespective of the season.

## Figures and Tables

**Table 1:** Baseline characteristics of the study group (*n* = 242).

Parameter	Total (*n* = 242)	Spring (*n* = 61)	Summer (*n* = 77)	Autumn (*n* = 64)	Winter (*n* = 40)	*p*
Female *n* (%)	172 (71.1)	39 (63.9)	57 (74.0)	49 (76.6)	27 (67.5)	0.38
Age (years)	78 (72–83)	80 (73–85)	78 (72–83)	78 (72–81)	77 (74–83.5)	0.38
BMI (kg/m^2^)	14 (24–31)	26 (23–30)	27 (24–30)	28 (25–32)	28 (25–34)	0.09
Length of hospitalized (days)	10 (8–12)	10 (8–14)	10 (8–11.5)	10 (8–12)	10 (9–12)	0.36
Arterial hypertension *n* (%)	198 (81.8)	48 (78.7)	60 (77.9)	54 (84.4)	36 (90.0)	0.35
Diabetes mellitus *n* (%)	85 (35.1)	16 (26.2)	31 (40.3)	20 (31.3)	18 (45.0)	0.16
Hypercholesterolemia *n* (%)	70 (28.9)	14 (23.0)	27 (35.1)	18 (28.1)	11 (27.5)	0.46
Hyperlipidemia *n* (%)	18 (7.4)	5 (8.2)	2 (2.6)	8 (12.5)	3 (7.5)	0.16
CAD *n* (%)	87 (36.0)	18 (29.5)	27 (35.1)	26 (40.6)	16 (40.0)	0.56
HF *n* (%)	63 (26.0)	19 (31.1)	20 (26.0)	14 (21.9)	19 (47.5)	**0.03**
Anemia *n* (%)	65 (26.9)	18 (29.5)	19 (24.7)	17 (26.6)	11 (27.5)	0.93
CKD *n* (%)	41 (16.9)	8 (13.1)	19 (24.7)	7 (10.9)	7 (17.5)	0.13
COPD *n* (%)	21 (8.7)	5 (8.2)	7 (9.1)	7 (10.9)	2 (5,0)	0.76
Stroke *n* (%)	23 (9.5)	7 (11.5)	8 (10.4)	6 (9.4)	2 (5.0)	0.73
Obesity *n* (%)	92 (38.0)	16 (26.2)	22 (28.6)	25 (39.1)	16 (40.0)	0.15
Overweight *n* (%)	79 (32.6)	21 (34.4)	33 (42.9)	24 (37.5)	14 (35.0)	0.15

**Abbreviations: BMI**—body mass index; **CAD**—coronary artery disease; **HF**—heart failure; **CKD**—chronic kidney disease; **COPD**—chronic obstructive pulmonary disease; arterial hypertension according to European Society of Hypertension/European Society of Cardiology (ESH/ESC) 2007 was recognized as SBP (systolic blood pressure): 140–159 (mmHg) and DBP (diastolic blood pressure): 90–99 (mmHg) [13].

**Table 2:** Serum 25(OH)D concentrations (nmol/L) across all seasons (*n* = 242).

	Median (Q1–Q3)	*p*
Spring	32.95 (25.96–43.68)	0.480
Summer	38.69 (27.46–50.67)	
Autumn	33.45 (27.08–44.18)	
Winter	34.57 (23.46–43.93)	
Total	33.95 (29.96–45.18)	

Q1, lower of quartile; Q3, upper of quartile.

**Table 3:** Status of serum 25(OH)D concentrations during seasons (*n* = 242).

	*n*	Deficiency 25(OH)D ≤50.0 nmol/L	Suboptimal 25(OH)D >50.0–75.0 nmol/L	Adequate 25(OH)D >75.0–125.0 nmol/L	*p*
		*n* (%)	*n* (%)	*n* (%)	0.297
Spring	61	51 (83.6)	9 (14.8)	1 (1.6)	
Summer	77	56 (72.7)	20 (26.0)	1 (1.3)	
Autumn	64	52 (81.2)	12 (18.8)	0 (0.0)	
Winter	40	34 (85.0)	5 (12.5)	1 (2.5)	
Total	242	193 (79.8)	46 (19.0)	3 (1.2)	

25(OH)D_3_, 25-hydroxyvitamin D_3._

**Table 4:** Median serum 25(OH)D concentrations (nmol/L) of patients hospitalized in each month (*n* = 242).

	*n* (%)	Median (Q1–Q3)	*p*
January	14 (5.8)	43.56 (23.71–50.67)	0.193
February	14 (5.8)	36.69 (25.71–43.68)	
March	17 (7.0)	30.45 (21.22–34.44)	
April	18 (7.4)	31.95 (27.21–46.68)	
May	21 (8.7)	32.70 (23.21–38.69)	
June	20 (8.3)	35.69 (27.46–49.42)	
July	28 (11.5)	42.18 (31.08–53.41)	
August	24 (9.9)	37.44 (27.46–50.54)	
September	30 (12.4)	33.45 (22.46–47.92)	
October	14 (5.8)	36.57 (28.45–49.92)	
November	22 (9.1)	30.58 (25.96–35.44)	
December	20 (8.3)	34.44 (28.70–46.80)	

Abbreviations: SD—standard deviation; Q1—lower of quartile; Q3—upper of quartile.

## Data Availability

All date are availability by address email of justyna.nowak@sum.edu.pl.
